# Flagella‐Mediated Biofilm Formation and 3D Swarming Drive Virulence in the Honey Bee Pathogen *Paenibacillus larvae*

**DOI:** 10.1111/1462-2920.70477

**Published:** 2026-11-03

**Authors:** Josefine Göbel, Anne Fünfhaus, Elke Genersch

**Affiliations:** ^1^ Department of Molecular Microbiology and Bee Diseases Institute for Bee Research Hohen Neuendorf Germany; ^2^ Department of Veterinary Medicine Institute of Microbiology and Epizootics, Freie Universität Berlin Berlin Germany

**Keywords:** American foulbrood, biofilm formation, flagella, *Paenibacillus larvae*, swarming motility

## Abstract

American foulbrood, a devastating bacterial disease affecting honey bee larvae, represents a major threat to apiculture worldwide. Its etiological agent, the Gram‐positive, endospore‐forming bacterium *Paenibacillus larvae* , has therefore been extensively studied to elucidate the molecular mechanisms underlying its pathogenicity and to identify key virulence determinants. In the present study, we identified the peritrichous flagella of *P. larvae* as a novel and important virulence factor of the genotype ERIC II. A flagella‐deficient mutant was generated and shown to exhibit multiple defects in vitro associated with biofilm formation, including altered biofilm architecture, reduced adhesion, impaired floating capacity and loss of swarming motility. Survival assays using honey bee larvae, complemented by fluorescence in situ hybridization (FISH) analyses of infected larvae, further demonstrated the crucial contribution of flagella to virulence in vivo by mediating biofilm formation on the larval midgut epithelium as well as three‐dimensional swarming during epithelial invasion.

## Graphical abstract

The flagella of the bacterial pathogen *Paenibacillus larvae* , the etiological agent of American foulbrood in honey bees, were characterized and identified as virulence factors. These cellular appendages play key roles in biofilm formation and 3D swarming, two multicellular behaviours of *P. larvae* that contribute to pathogenesis.

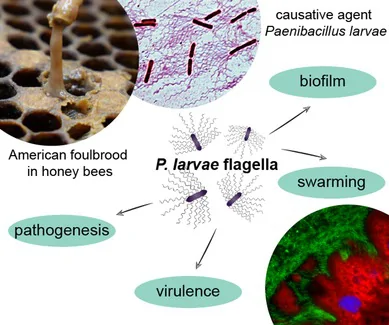

## 1 Introduction

Human food security depends to a large extent on plant‐based foods, including fruits and oilseeds, whose yields largely depend on pollination by insects. Among the pollinating insects, honey bees are the most important commercial pollinators mainly because they are managed by humans and can therefore be specifically transported and deployed where their pollination service is needed (Morse and Calderon 2000; Aizen and Harder 2009; Papa et al. 2022). For high pollination performance, strong and healthy colonies are essential. However, like all other living creatures, honey bees are threatened by a broad range of metazoan parasites as well as viral, fungal and bacterial pathogens, all of which can impair the health and survival of individual bees and may ultimately lead to colony losses, thus negatively affecting pollination as agro‐ and ecosystem services worldwide (Genersch 2010b; Cornman et al. 2012; Meixner et al. 2014).

The most dangerous bacterial pathogen affecting honey bees is the spore‐forming bacterium *Paenibacillus larvae* , whose spores are infectious—though only to young honey bee larvae—and which causes the deadly brood disease American foulbrood (AFB) (Hoage and Rothenbuhler 1966; Genersch et al. 2006). Although *P. larvae* affects only the larval stages, the death of the diseased larvae results in the increasing decline or loss of the subsequent generations of worker bees and ultimately to the collapse of the colony (Ebeling et al. 2016). AFB is not only a lethal disease, but it is also highly contagious as the infectious spores can easily be transmitted within and between colonies (Lindström et al. 2008a, 2008b). Therefore, it is classified as a notifiable epizootic in numerous countries and measures to contain the disease are often regulated by law. While infected, not yet diseased colonies can be cleansed from AFB by the so‐called shook swarm method, diseased colonies are usually required to be culled (Hansen and Brodsgaard 2003; Pernal et al. 2008). This situation presents beekeepers with a true dilemma, as diseased colonies either die or are culled and are therefore lost in any case. Besides the use of antibiotics, which is prohibited in many countries, no sustainable treatment strategies against AFB exist. To have a chance to develop these, a better understanding of the pathomechanisms and virulence factors of *P. larvae* is urgently needed.

*P. larvae* is a rod‐shaped, Gram‐positive bacterium, which is capable of forming highly tenacious spores that represent the infectious and easily transmittable form of the bacterium (Haseman 1961; Genersch et al. 2006; Lodesani et al. 2015). The ability of *P. larvae* to metabolize the sugary larval food, but also larval structures like the peritrophic matrix, and eventually the larval tissues renders honey bee larvae an extremely nutrient‐rich niche for these bacteria (Neuendorf et al. 2004; Yue et al. 2008; Garcia‐Gonzalez and Genersch 2013). The pathogen can initiate an infection in the larval midgut when young larvae under the age of 36 h after egg hatching ingest spores via contaminated food (Hoage and Rothenbuhler 1966; Davidson 1970). The spores germinate in the larval midgut, where the vegetative bacteria initially thrive on the continuously incoming larval food and colonize the entire larval midgut (Neuendorf et al. 2004; Yue et al. 2008). This commensal‐like phase is followed by an invasive phase, in which the bacteria attack and breach the midgut epithelium using the paracellular route to invade the larval hemocoel; this step in AFB pathogenesis is accompanied by the death of the host (Yue et al. 2008). Death of the host is not collateral damage, but rather the interim aim of the obligate killer *P. larvae* , that now switches to a necrotrophic life style and uses the larval carcass as a nutrient source thus totally decomposing the dead larva to a ropy mass. Shortage of nutrients presumably initiates sporulation so that the ropy mass, when dried into the so‐called AFB scale, contains billions of *P. larvae* spores (Genersch 2010a; Poppinga and Genersch 2015).

Two genotypes of *P. larvae* are currently responsible for the worldwide occurrence of AFB outbreaks: *P. larvae* ERIC I and ERIC II. These are classified by repetitive element PCR (rep‐PCR) using *enterobacterial repetitive intergenic consensus* (ERIC) primers and exhibit differences in morphology, metabolism and, most notably, virulence (Neuendorf et al. 2004; Genersch et al. 2006). Intensive research has identified a number of different virulence factors, some of which are genotype‐specific, which have shed light on the virulence strategies of *P. larvae* . A key virulence factor of the species *P. larvae* is the chitinase *Pl*CBP49 that is capable of degrading the chitin‐rich peritrophic matrix normally protecting the midgut epithelium against pathogen attack (Garcia‐Gonzalez and Genersch 2013; Garcia‐Gonzalez et al. 2014). For the subsequent breaching of the unprotected gut epithelium and the invasion of the hemocoel, the genotypes *P. larvae* ERIC I and ERIC II have different virulence factors at their disposal. *P. larvae* ERIC I attacks the epithelial cells with two ADP‐ribosylating AB‐toxins, Plx1 and Plx2 (Fünfhaus et al. 2013; Ebeling et al. 2017, 2021), whose genes are no longer functional in the genome of *P. larvae* ERIC II due to various mutations (Djukic et al. 2014). The damage to the epithelial cells caused by Plx1 and Plx2 then enables *P. larvae* ERIC I to penetrate the epithelium via the paracellular route. In contrast, *P. larvae* ERIC II lacking any toxins (Djukic et al. 2014; Ebeling et al. 2019) expresses a surface‐layer protein, SplA, whose gene is nonfunctional in *P. larvae* ERIC I due to a frameshift mutation (Poppinga et al. 2012). SplA mediates the adhesion of *P. larvae* ERIC II to the midgut epithelium, a step that is considered necessary for the subsequent breach of the epithelium and invasion of the hemocoel (Poppinga et al. 2012), which is most likely facilitated by the simultaneous production of extracellular proteases (Holst and Sturtevant 1940; Dancer and Chantawannakul 1997; Antunez et al. 2009, 2011).

The entire species *P. larvae* was described from the outset as a peritrichously flagellated bacterium (White 1920), and the flagella shed during sporulation form so‐called giant whips, which serve as a diagnostic feature for the identification of *P. larvae* in the laboratory (Plagemann 1985). Flagella are helical, thread‐like appendages of bacterial cells with a complex structure comprising roughly three architectural domains: the membrane‐anchored basal body (including a secretion apparatus and a motor), the hook and the extracellular filament (Mukherjee and Kearns 2014). Flagella can generate propeller‐like movements thanks to their integrated motor, thus providing the driving force for often directed multicellular motility, that is, swimming as a population or swarming across a surface (Henrichsen 1972; Jones et al. 2004; Copeland et al. 2010). However, flagella are not only used by bacteria for motility, but according to recent reports are also involved in another multicellular behaviour, namely the formation of biofilms. During biofilm formation they can function as adhesin or autoagglutinin, mediating the binding to a surface or inducing bacterial autoaggregation, respectively (Haiko and Westerlund‐Wikström 2013; Trunk et al. 2018). Furthermore, bacterial flagella can pose a structuring component of the extracellular matrix (ECM) within the biofilm (Liu et al. 2019; Ozer et al. 2021).

Considering that *P. larvae* is peritrichously flagellated (White 1920), it is not surprising that it has recently been shown that this species is capable of multicellular cooperative behaviour (Fünfhaus et al. 2018). Both *P. larvae* ERIC I and ERIC II were able to form biofilms. Biofilm cells were embedded in a self‐produced ECM and formed submerged but free‐floating biofilms that loosely adhered to the walls of the culture wells. Interestingly, only *P. larvae* ERIC II, but not *P. larvae* ERIC I, exhibited swarming motility on the surface of agar plates, suggesting that the ability to swarm is another characteristic that distinguishes these two genotypes (Fünfhaus et al. 2018). This study also demonstrated that the production of paenilarvin, an iturin‐like secondary metabolite produced specifically by *P. larvae* ERIC II, was involved in the observed swarming motility. It was postulated that the formation into cooperative cell communities could be important for *P. larvae* during early stages of infection as well as during the necrotrophic phase (Fünfhaus et al. 2018).

Given their multifaceted roles, including their involvement in cooperative behaviours like swarming and biofilm formation, bacterial flagella are frequently linked to the virulence of pathogenic bacteria and can play a crucial role in pathogen‐host interactions (Duan et al. 2013; Haiko and Westerlund‐Wikström 2013). In this study, we sought to elucidate the role of flagella in the multicellular behaviours exhibited by the honey bee pathogen *P. larvae* ERIC II and, consequently, in the virulence of the bacterium. To this end, we searched the annotated genome of *P. larvae* ERIC II (DSM25430) (Djukic et al. 2014) and identified four gene clusters involved in flagella biosynthesis and identified the gene encoding the flagellar filament structural protein (flagellin) through *in silico* analysis of the flagella biosynthesis genes in the genome of *P. larvae* DSM25430. We then constructed a gene‐inactivation mutant that was deficient in the production of flagellin, resulting in a bald phenotype. The *P. larvae* mutant was tested in vitro in swarming as well as in biofilm assays and in vivo using exposure bioassays with honey bee larvae as the host organism. Analysis of infected larvae with fluorescence in situ hybridization (FISH) uncovered fundamental roles for biofilm formation and collective motility of *P. larvae* ERIC II during infection. Our results show that flagella are essential for the full virulence of this *P. larvae* genotype. The identification of flagella as a new virulence factor of *P. larvae* ERIC II expands our understanding of AFB pathogenesis.

## 2 Material and Methods

### 2.1 Bacterial Strains and Growth Conditions

In the course of this study, the strain DSM25430 (Leibnitz Institute DSMZ—German Collection of Microorganisms and Cell Cultures GmbH) was utilized as representative for the *P. larvae* genotype ERIC II. This strain corresponds to the field isolate 04‐309 from a foulbrood outbreak in Germany, which has been comprehensively examined in numerous studies and corresponds to the specific genotype (ERIC II) with regard to its virulence and phenotypic characteristics (Genersch et al. 2005, 2006; Fünfhaus et al. 2009; Rauch et al. 2009; Fünfhaus and Genersch 2012). *P. larvae* was cultivated on Columbia sheep blood agar (CSA) plates (Oxoid Limited, Hampshire, UK) at 37°C for 2–3 days, unless otherwise specified. For liquid cultures of *P. larvae*, Brain Heart Infusion Broth (BHI; Merck KGaA, Darmstadt, Germany), MYPGP medium (Dingman and Stahly 1983) or Sf900 SFM II medium (Thermo Fisher Scientific/Life Technologies, Darmstadt, Germany) were used, with shaking incubation, as described in detail for the respective assays. Long‐term storage of all *P. larvae* strains was carried out in glycerol stocks at −70°C.

*P. larvae* spore suspensions were generated as previously described (Genersch et al. 2005; Poppinga et al. 2012). Briefly, approximately 100 bacterial colonies were resuspended in 300 μL of BHI to inoculate the liquid portion of CSA slants. These were then incubated at 37°C for a period of at least 10 days. Spore concentrations were estimated by cultivating serial dilutions on CSA plates and determining the colony forming units per mL (cfu/mL). The DNA of *P. larvae* was extracted to be used in the conducted PCR analyses according the following procedure: One inoculation loop of bacteria was resuspended in 50 μL of water and subsequently heated at 95°C. Following centrifugation at 5000 *g* for 5 min, the DNA was found to be present in the supernatant.

*Escherichia coli* DH5α cells that had been transformed with the plasmid pTT_flaA_79 (see below) were cultivated in selective Luria Bertani (LB) media (agar and broth) supplemented with 30 μg/mL chloramphenicol. Plasmid DNA preparations were carried out following the manufacturer's protocol (QIAprep Spin Miniprep kit; Qiagen, Hilden, Germany).

### 2.2 Gene Cluster Analysis

The genome sequence of *P. larvae* DSM25430 (NCBI reference sequence NC_023134.1), which had already been deposited at the National Center for Biotechnology Information (NCBI), was utilized to analyse the genomic structure of the flagella biosynthesis genes of *P. larvae* ERIC II. The determination of the genomic structure, gene lengths and protein masses was conducted using the SnapGene 7.0.2 software. The prediction of protein products was performed using the protein BLAST tool of NCBI (Sayers et al. 2025), the UniProt database (UniProt 2025) as well as InterProScan (Jones et al. 2014). The graphic of the genomic structure of flagella biogenesis genes of *P. larvae* was created with Adobe Illustrator 29.0.

### 2.3 Construction of a *P. larvae fla*A Gene‐Inactivation Mutant

The gene *fla*A in *P. larvae* DSM25430 was inactivated through the targeted insertion of a group II intron into the respective open reading frame. This method, which has been previously established for *P. larvae* (Poppinga et al. 2012) was based on the ‘TargeTron Gene Knockout System’ (Sigma‐Aldrich/Merck KGaA, Darmstadt, Germany) and was modified by Zarschler et al. (2009). To this end, the vector‐localized intron RNA was modified using specific primers (Table 1) so that the Ll.LtrB intron (from *Lactococcus lactis* ) would insert specifically after 79 bp in the *fla*A gene, starting from the start codon, thereby disrupting the reading frame of the gene. The modified vector pTT_flaA_79 was initially introduced into chemocompetent *E. coli* DH5α cells for plasmid replication and isolation. Additional sequencing was performed to verify the intron's mutagenesis (see Table 1 for primer sequences). Thereafter, wild‐type *P. larvae* bacteria (DSM25430) were transformed with the corresponding plasmid by means of electroporation. The production of electrocompetent *P. larvae* cells and the subsequent transformation was performed as previously described (Poppinga and Genersch 2012). Transformed cells were then cultured on a selective medium (MYPGP agar with 5 μg/mL chloramphenicol) until the LI.LtrB intron had successfully integrated into the target gene. The verification of this integration was conducted through PCR analysis, employing primers flanking the insertion sites (Table 1; Figure S1). In order to ascertain that the intron insertion in *fla*A would not impede the growth of *P. larvae* and that the constructed knockout mutant would therefore be suitable for subsequent assays, growth rates of the wild type and mutant strain were assessed as follows: Precultures of three biological replicates were prepared by inoculating MYPGP medium with one loop full of bacteria and shaking incubation over night at 37°C. The cultures were subsequently diluted to an OD600 of 0.001, after which 200 μL of the diluted cultures were transferred in triplicate to a 96‐well plate (Corning3603, Merck KGaA, Darmstadt, Germany). Wells containing only MYPGP medium served as blanks. The plates were subjected to agitation at 37°C, and the OD600 was measured at hourly intervals (Synergy HT, BioTek/Agilent Technologies, Waldbronn, Germany) until the stationary phase was attained (the same samples were measured repeatedly). The experiment was repeated three times.

**TABLE 1:** Primer used for the construction and verification of the gene inactivation in *Paenibacillus larvae* DSM25430 (ERIC II).

Primer name	Primer sequences	Used for
IBS1/2_flaA_79	5′‐AAAAAAGCTTATAATTATCCTTAACAGCCTTTCCAGTGCGCCCAGATAGGGTG −3′	knockout construction
EBS1/delta_flaA_79	5′‐CAGATTGTACAAATGTGGTGATAACAGATAAGTCTTTCCATGTAACTTACCTTTCTTTGT‐3′	
EBS2_flaA_79	5′‐TGAACGCAAGTTTCTAATTTCGATTGCTGTTCGATAGAGGAAAGTGTCT‐3′	
pTT‐TT‐Intron_F	5′‐GTCTTGGTAAATTCAGATTCTCGG‐3′	Sequencing of retargeted intron in vector pTT
pTT_TT‐Intron_R	5′‐AGGCAATGAATCTACTAAACGG‐3′	
Flag_F3	5′‐ATGAACACGCATCGCAAC‐3′	Screening for intron insertion
Flag_R3	5′‐AAGCAATTGAAGCACGCC‐3′	

To verify the *fla*A gene inactivation via protein analysis, wild‐type *P. larvae* and knockout mutant *P. larvae* were cultivated in 5 mL of BHI overnight at 37°C. Subsequently, 2 mL of each culture was transferred to an Eppendorf reaction tube and subjected to centrifugation (10 min, 8000*g*) to harvest the cells. The bacterial pellets were then subjected to sodium dodecyl sulphate polyacrylamide gel electrophoresis (SDS‐PAGE) to assess the production of the flagellin protein (Laemmli 1970). See Figure S2 for a full‐height image of SDS‐PAGE.

### 2.4 Visualization of Flagella

The staining of the flagella of *P. larvae* was carried out according to the method described by Leifson (Leifson 1951) at three different time points of *P. larvae* growth. For this purpose, precultures of wild‐type *P. larvae* and the ∆*fla*A mutant were prepared by inoculating 2 mL of BHI with one inoculation loop of bacteria, followed by shaking incubation overnight at 37°C. The precultures were then diluted to an OD_600_ of 0.01 in 5 mL of BHI and incubated at 37°C with shaking. Subsequently, 500 μL solution was collected from the upper portion of the culture (no prior vortexing) after (I) 17 h, (II) 24 h and (III) 48 h of incubation and transferred to an Eppendorf reaction tube. The three time points correspond, respectively, (I) to the exponential phase, (II) the late exponential phase and (III) the stationary phase of *P. larvae* ERIC II growth. The bacterial samples were subjected to centrifugation (5 min, 5000*g*) to pellet the cells, followed by a single wash in sterile MilliQ water. The samples were then resuspended in MilliQ water. A smear was prepared on a clean slide and dried at room temperature. The smear was subsequently covered with the Leifson staining solution. This staining solution was prepared by combining three solutions in equal parts: 1.5% NaCl, 3% tannic acid and 1.2% basic fuchsin in ethanol. Following an incubation period of 5 min with the staining solution, the slide was washed with MilliQ water and dried once more at room temperature. A coverslip was affixed to the slide using a drop of Enthellan (Merck KGaA, Darmstadt, Germany). The stained flagella were then analysed at 600× magnification with oil using brightfield microscopy (Eclipse Ti, Nikon GmbH, Düsseldorf, Germany).

### 2.5 Whole‐Genome Sequencing of the *P. larvae* Mutant DSM25430 ∆ *fla*A

The genome of the constructed *P. larvae* mutant DSM25430 Δ*fla*A was subjected to sequencing using Oxford Nanopore Technologies (ONT). For this purpose, 5 mL of MYPGP medium were inoculated with one inoculation loop of DSM25430 Δ*fla*A bacteria and incubated overnight at 37°C with agitation to obtain precultures. Subsequently, 1 mL of preculture was transferred to 10 mL of 2× MYPGP medium, and the suspension was incubated for 24 h at 37°C with agitation. The cells were harvested by centrifugation at 3220 *g* for 20 min at 4°C. Bacterial DNA was isolated using the Monarch HMW DNA Extraction Kit for Tissue (New England Biolabs, Ipswich, MA, USA) according to the manufacturer's protocol. Subsequent to this, ONT bacterial genome sequencing was performed by Eurofins Genomics (Ebersberg, Germany). This genome has been deposited at NCBI under the GenBank accession number JCFKJT000000000.1 (NCBI BioProject PRJNA1516485). The version described in this paper is version JCFKJT010000000. The genome sequence of the *P. larvae* mutant was comprehensively analysed using Geneious 9.1.8, including identification of the intron insertion site and exclusion of alterations in the *spl*A gene and the paenilarvin gene cluster.

### 2.6 Swarming Assay

Swarming assays were performed in accordance with the protocol previously established (Fünfhaus et al. 2018). The required swarming plates were cast from 0.5% BHI agar, which was cooled in a 60°C water bath for 30 min after melting. The plates were then left to dry for 15 min with the lid open. This specific procedure controlled the water content of the agar plates, which is a critical factor in the swarming of bacteria (Kearns 2010). Precultures of *P. larvae* , cultivated at 35°C overnight in BHI broth with agitation, were diluted to an OD_600_ of 0.1. A total of 10 μL of the dilutions was meticulously deposited into the centre of each agar plate. The plates were then allowed to dry for an additional 15 min with the lid open until the cultures were fully absorbed into the agar. Thereafter, the plates were placed in an incubator maintained at 37°C. After 2 and 3 days, the spread of the bacteria on the swarm plates was observed. The experiment was repeated three times with three biological replicates each. Images of the swarm plates were taken in a MultiImage Light Cabinet (Alpha Innotech Corporation, San Leandro, USA).

### 2.7 Congo Red Biofilm Assay

The cultivation and Congo red staining of *P. larvae* biofilms were performed as previously described (Fünfhaus et al. 2018). Precultures of the wild‐type *P. larvae* strain DSM25430 wt and the corresponding knockout mutant DSM25430 ∆*fla*A were prepared in 2 mL BHI and incubated overnight at 37°C with shaking. A 6‐mL bacterial suspension with an OD_600_ of 0.1 was prepared from each preculture and transferred to the well of a 6‐well cell culture plate (Greiner Bio‐One GmbH, Frickenhausen, Germany). BHI alone was added to the wells of the negative control. The well plates were sealed with Parafilm to prevent evaporation and then incubated for 5 days at 37°C. Images of the unstained cultures were captured using a MultiImage Light Cabinet (Alpha Innotech Corporation, San Leandro, USA). Subsequently, Congo red (Merck KgaA, Darmstadt, Germany) was added to each culture at a final concentration of 30 μg/mL. The stained biofilms were then imaged after a 24‐h static incubation at 37°C (Olympus CAMEDIA C‐5060, Olympus Corporation, Tokyo, Japan). The assay was repeated three times, with each assay comprising three biological replicates.

The binding of Congo red by the ECM of *P. larvae* DSM25430 wildtype and the mutant strain DSM25430 Δ*fla*A was measured as previously described (Janesch et al. 2013; Fünfhaus et al. 2018). For this purpose, 1 mL of statically grown, 5‐day‐old bacterial culture was incubated together with 30 μg of Congo red at 37°C with shaking (300 rpm) for a period of 2 h. Subsequently, the Congo red that had bound to the matrix of the *P. larvae* biofilms was removed by means of centrifugation (5 min, 13,000*g*). The amount of unbound Congo red was then quantified by measuring the absorbance of the resultant supernatant at 490 nm (Synergy HT microplate reader, BioTek, Winooski, USA). BHI medium, which was concomitantly incubated with Congo red, was utilized as a reference value for calculating the amount of bound Congo red. Three assays were performed, with each assay involving three biological replicates.

### 2.8 Crystal Violet Biofilm Assay

In order to visualize biomass of *P. larvae* adherent to the walls of the culture wells, staining with crystal violet was performed. This staining method is well‐established for its use in static biofilms (Merritt et al. 2005) and has previously been used for the detection of *P. larvae* biofilms (Fünfhaus et al. 2018). For this purpose, *P. larvae* was cultivated overnight at 37°C in BHI with agitation and subsequently diluted to an OD_600_ of 0.1 the following day. A volume of 150 μL of the diluted bacterial cultures was pipetted into the wells of a 96‐strip well plate (Greiner Bio‐One GmbH, Frickenhausen, Germany). The same amount of BHI medium was used as a negative control. The remaining wells of the plate were filled with water. The plate was then subjected to static incubation at 37°C for a period of 6 days. Subsequently, the plate was washed thrice with MilliQ water to ensure the removal of unbound cells. For the staining of bound cells, 200 μL of 0.1% crystal violet was added to each well. After a 5 min incubation period with the dye, it was tilted off and the plate was washed thrice more in water prior to image capture (Olympus CAMEDIA C‐5060, Olympus Corporation, Tokyo, Japan). To quantify the bound cell mass, the crystal violet stain was dissolved in 200 μL of 95% ethanol (95%) per well. Then 100 μL of the solution was transferred to a new 96‐well plate, and the solution's absorbance was measured at 570 nm (Synergy HT, BioTek/Agilent Technologies, Waldbronn, Germany). The staining of the negative control was used as a blank. The assay was performed at least three times with three biological and three technical replicates each.

### 2.9 Thioflavin S Biofilm Assay

To investigate the effect of the *fla*A knockout on the three‐dimensional structure of the biofilm of *P. larvae* , the ECM was visualized using the amyloid‐binding dye Thioflavin S, as previously described (Fünfhaus et al. 2018). The experimental setup commenced with the inoculation of 2 mL of Sf900 SFM II medium (Thermo Fisher Scientific/Life Technologies, Darmstadt, Germany) with one inoculation loop of bacteria, followed by an overnight incubation at 37°C to yield precultures of *P. larvae* . From these cultures, dilutions with an OD_600_ of 0.01 were prepared, of which 100 μL each were transferred into the wells of a 96‐well plate (Greiner Bio‐One GmbH, Frickenhausen, Germany). To each well, 30 μg/mL Thioflavin S (Sigma‐Aldrich/Merck KGaA, Darmstadt, Germany) was added, while negative controls contained only medium and Thioflavin S. Empty wells of the plate were filled with water. Plates were sealed with Parafilm and incubated at 37°C under static conditions for 6 days. Biofilm‐bound Thioflavin S was visualized via fluorescence microscopy (Eclipse Ti, Nikon GmbH). Z‐stacks were acquired to generate 3D images (NIS‐Elements AR 3.1). In a series of three experiments, each comprising three biological replicates, the mean vertical spread of the biofilm matrix within each replicate was evaluated by measuring the expansion of thioflavin S fluorescence at six distinct locations within the respective biofilm. Orthogonal projections of the image stacks were used to obtain the measurements (NIS‐Elements AR 3.1).

### 2.10 Bioassays With Honey Bee Larvae

In order to investigate the effects of the *fla*A gene inactivation on the pathogenicity of *P. larvae* ERIC II, laboratory infection experiments with honey bee larvae were carried out, as previously described (Genersch et al. 2005, 2006; Crailsheim et al. 2013). Larvae for laboratory infection experiments were collected from honey bee colonies ( *Apis mellifera carnica* ) kept at the institute's apiary. To minimize the influence of the larval genetic background on the outcome of *P. larvae* infections, first instar larvae were grafted from at least 10 and up to 20 colonies not headed by sister or genetically related queens to ensure a mixed genetic background of the tested larvae (Crailsheim et al. 2013), transferred into 24‐well plates (initially 10 larvae per well) and exposed to larval diet (66% royal jelly (vol/vol) (Werner Seip, Butzbach‐Ebersgöns, Germany), 3% glucose (wt/vol) and 3% fructose (wt/vol)) spiked with spores of wild‐type *P. larvae* DSM25430 wt or of the gene‐inactivation mutant *P. larvae* DSM25430 ∆*fla*A at a final concentration of 300 cfu/mL. After 24‐h, the larvae were transferred to food devoid of spores, which was replenished on a daily basis (300 μL/well) for the duration of the experiment. Control groups received exclusively spore‐free food. As the larvae increased in size, the number of larvae kept per well was progressively reduced until there was ultimately one larva per well (Figure S3). Once the larvae had completed the feeding process and initiated defecation, they were transferred to wells lined with paper towels, where the pupal phase ensued.

The larvae were reared in a controlled environment, with temperatures maintained at 35°C and a constant absence of light. Daily assessment was conducted for a period of 14 days following infection to determine survival rates. Dead larvae were removed and spread on CSA plates to verify an infection with *P. larvae* as the cause of death. The identification of *P. larvae* was conducted through phenotypical analysis and PCR assay, employing *P. larvae* ‐specific primers designed for knockout screening (Table 1) which also confirmed intron insertion stability. The experiment was repeated four times with three groups per run (control, wild type, gene inactivation), each comprising 30 larvae, giving a total number of 120 larvae per group. *P. larvae* was never detected in control larvae and the natural mortality did not exceed an average ratio of 15% in any of the groups (Crailsheim et al. 2013).

### 2.11 FISH Analysis of Larvae Infected With *P. larvae*

To analyse the behaviour of *P. larvae* during infection of honey bee larvae, FISH was performed with honey bee larvae infected with either DSM25430 wt or DSM25430 Δ*fla*A or with noninfected control larvae essentially as previously described (Yue et al. 2008). From all groups, live larvae were collected from the 24‐well plate at varying time points postinfection and were subsequently preserved in 4% formaldehyde in PBS (pH 7.5). Larvae were then embedded in HistoCure 8100 (Morphisto, Main, Germany), followed by blocking up with Technovit 3040 (Morphisto, Main, Germany) according to the manufacturer's instructions. Histological sections (5 μm) were prepared with a microtome (MICROM HM355S; Thermo Scientific) using steel knives with tungsten carbide edges. Sections were mounted on HistoBond adhesive microscope slides (Paul Marienfeld GmbH & Co. KG, Lauda‐Königshofen, Germany) with the help of sterile water for straightening, followed by drying on a slide warmer. Fluorescence‐labelled FISH probes were synthesized commercially (Eurofins Genomics, Ebersberg, Germany). The species‐specific *P. larvae* probe ( *P. larvae* 16S; 5′‐CTAACCTGGCGTCTCCCGAA‐3′) was designed according to Yue et al. (2008), labelled with Carboxytetramethylrhodamine (TAMRA) at both ends and used to visualize vegetative *P. larvae* bacteria. The probe EUK516 (5′‐ACCAGACTTGCCCTCC‐3′), complementary to a unique target site on the 18S rRNA of many eukaryotes (Amann et al. 1990), was labelled with Cy5 at the 5′‐end and used to visualize the honey bee larval cells. Prior to hybridization, the slides were washed with 1 × PBS, followed by an incubation with Proteinase K (1 μg/mL) for 10 min at 37°C to improve permeability of the sections. Subsequently, the hybridization of the aforementioned probes was conducted in a humid chamber at 45°C overnight, employing a final concentration of 50 ng/μL of each probe that had been diluted in 25 μL of hybridization buffer composed of 20% (v/v) formamide, 0.9 M NaCl, 20 mM Tris–HCl pH 7.9 and 0.01% (wt/vol) SDS for each section. Subsequently, the slides were thoroughly washed in 1 × PBS and dried at room temperature. Thereafter, each section was incubated with 100 μL of Quenching Solution (Vector TrueVIEW Autofluorescence Quenching Kit, Vector Laboratories, Newmark, United States) for 7 min, followed by a final round of washing and drying at room temperature. Cover slides were then fixed with 10 μL of Antifade Mounting Medium with DAPI (Vector TrueVIEW Autofluorescence Quenching Kit) for concurrent staining of eukaryotic nuclei. Probe signals and DAPI staining were then visualized using fluorescent microscopy (Eclipse Ti, Nikon).

### 2.12 Statistics

All statistical analyses were performed using GraphPad Prism 9.5.0. Growth curves were analysed using a repeated measures two‐way ANOVA with Bonferroni post hoc correction. Biofilm data are presented as mean ± standard deviation, or standard error of the mean, as indicated in the figure captions. Bar charts are overlaid with individual data points. Statistical comparisons between two groups were performed using an unpaired Student's *t* test (two‐tailed) after assessing normality using the Shapiro–Wilk test. Survival data of larvae from four infection experiments were analysed at the level of individual larvae using Kaplan–Meier survival analysis, followed by pairwise comparison of infection groups using the log‐rank (Mantel‐Cox) test. The hazard ratio (HR) was estimated using Cox proportional hazards regression. Death due to *P. larvae* infection was defined as the event, while larvae that died without evidence of infection were treated as censored observations. *p* < 0.05 were considered significant, with levels of significance indicated as: *p* ≤ 0.001***. The sample size for each experiment, as well as the number of replicates, is presented in the corresponding method sections and figure legends.

## 3 Results

### 3.1 Flagella Biosynthesis Gene Clusters

In the genome of *P. larvae* ERIC II (DSM25430; NCBI acc. no. NC_023134.1) (Djukic et al. 2014) 49 annotated genes were assigned to the biosynthesis of the flagellar apparatus (Table S1). Our *in silico* analysis revealed that these genes are organized into four clusters (Figure 1A), resembling the organization of the gene clusters for flagellum biosynthesis in *Bacillus subtilis* (Mukherjee and Kearns 2014) (Figure 1B). The largest cluster (ref. seq. NC_023134.1, pos. 2,164,135–2,193,919) is 29 kb long and highly similar to the 27‐kb long fla/che‐operon of flagellar and chemotaxis genes in *B. subtilis* (Mukherjee and Kearns 2014). The *P. larvae* fla/che cluster is responsible for the synthesis of the hook‐basal body complex of the flagellum, including the secretion system, the motor, the rod and the hook (Figure 1A). It also harbours six chemotaxis genes (*che*Y, *cheA–D, che*W) and four genes for regulatory proteins (*swr*D, *flh*F, *flh*G, *sig*D, *fap*A) (Figure 1A). The fla/che clusters of *P. larvae* and *B. subtilis* differ in only three genes: In contrast to *B. subtilis* , the *P. larvae* fla/che cluster encodes an additional basal body protein, FlgG, a hypothetical protein between CheD and SigD, and FapA instead of SwrB at the 3′‐end of the operon. Farther downstream, two dicistrons were identified, one (ref. seq. NC_023134.1, pos. 70,767–72,529; Figure 1A) harbouring two further basal body protein‐encoding genes, designated *flh*O and *flh*P and the other one (ref. seq. NC_023134.1, pos. 138,737–140,282; Figure 1A) harbouring the genes *mot*A and *mot*B, which encode the two stator proteins of the flagellar motor. The fourth cluster (ref. seq. NC_023134.1, pos. 103,461–112,493; Figure 1A) comprises genes necessary for the assembly of the *P. larvae* flagellar filament, which therefore represent the end of the transcriptional hierarchy during the synthesis of the bacterial flagellum (Aldridge and Hughes 2002; Mukherjee and Kearns 2014). This cluster consists of 12 annotated genes (Figure 1A) and includes the gene encoding the filament structural protein flagellin, which is referred to here as *fla*A. The genomic organization of the two dicistrons and the 9 kb‐long cluster is again analogous to *B. subtilis* (Figure 1B) (Mukherjee and Kearns 2014), with the exception of the genes *yvy*C and *smi*A, which are absent from the 9 kb‐cluster in *P. larvae* ERIC II.

**FIGURE 1:**
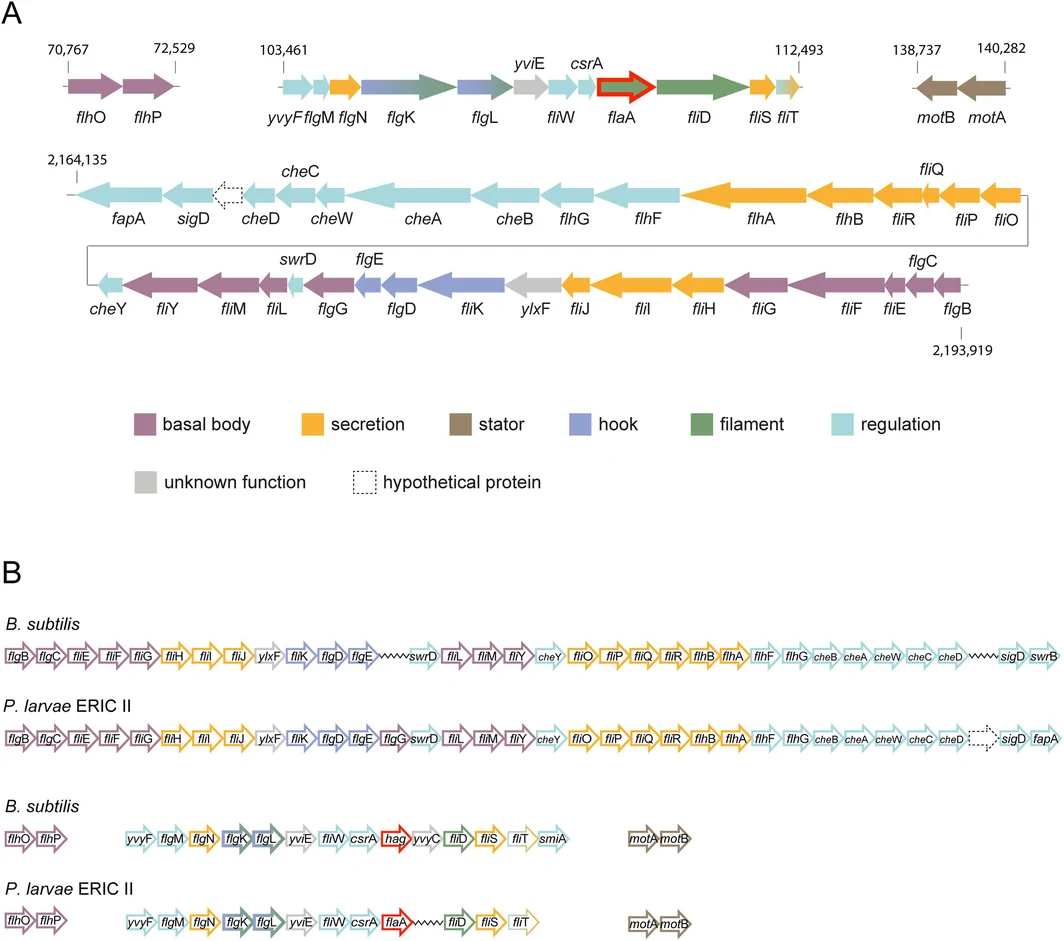
Gene clusters encoding the genes for the biosynthesis of flagella in *Paenibacillus larvae* ERIC II. (A) In the genome of *P. larvae* ERIC II (DSM25430; NCBI acc. no. NC_023134.1), 49 genes were identified as being involved in the biosynthesis of the flagellar apparatus in the *P. larvae* ERIC II genome. The genes are organized into four clusters, with arrows representing genes that are colour‐coded according to the function of their synthesis product. Numbers indicate the positions at the beginning and end of each cluster according to the NCBI reference sequence NC_023134.1. (B) Comparison of flagellar biosynthesis gene clusters in *Bacillus subtilis* (Mukherjee and Kearns 2014; Sanchez et al. 2022) and *P. larvae* ERIC II. The flagellin‐encoding gene, which was inactivated in *P. larvae* in this study, is highlighted with a red outline.

Some bacterial species have been observed to possess multiple flagellin‐encoding genes (Guerry et al. 1991; Bonifield and Hughes 2003; Jung et al. 2019). However, no other flagellin‐encoding gene besides *fla*A was detected in the *P. larvae* genome. Consequently, *fla*A was identified as a suitable target for generating a flagellum‐deficient gene‐inactivation mutant of *P. larvae* ERIC II, which would help elucidate the function of flagella during the pathogenesis of *P. larvae* infections.

### 3.2 Inactivation of *fla*A Results in a Flagella‐Deficient, Bald Phenotype of *P. larvae*

To investigate the role of *P. larvae* flagella during the pathogenesis of *P. larvae* infections, a *P. larvae* ERIC II mutant deficient in flagellin expression (DSM25430 ∆*fla*A) was constructed. This was accomplished by using the TargeTron system (Sigma‐Aldrich) in combination with a modified vector (Zarschler et al. 2009) to insert a group II intron into the 858 bp flagellin gene *fla*A of *P. larvae* DSM25430. To confirm the successful inactivation of *fla*A gene expression and the resulting disruption of flagellin protein synthesis, a protein analysis was performed on the gene‐inactivation mutant DSM25430 ∆*fla*A. Proteins from the bacterial fraction of overnight cultures of DSM25430 wild‐type bacteria (DSM25430 wt) and DSM25430 ∆*fla*A were separated by sodium dodecyl sulphate‐polyacrylamide gel electrophoresis (SDS‐PAGE). While the protein flagellin (FlaA; 31.3 kDa) appeared as a dominant band within the protein band pattern of DSM25430 wt, this band was absent in the corresponding gene‐inactivation mutant DSM25430 ∆*fla*A (Figure 2A). Therefore, it can be concluded that the production of flagellin was successfully disrupted by the intron insertion into *fla*A. No further changes were observed in the protein banding pattern of the mutant compared with that of the wild‐type bacteria; in particular, the expression of the S‐layer protein SplA—another protein involved in the adhesion of *P. larvae* to epithelial cells (Poppinga et al. 2012)—appears unaffected in the mutant (Figure 2A).

**FIGURE 2:**
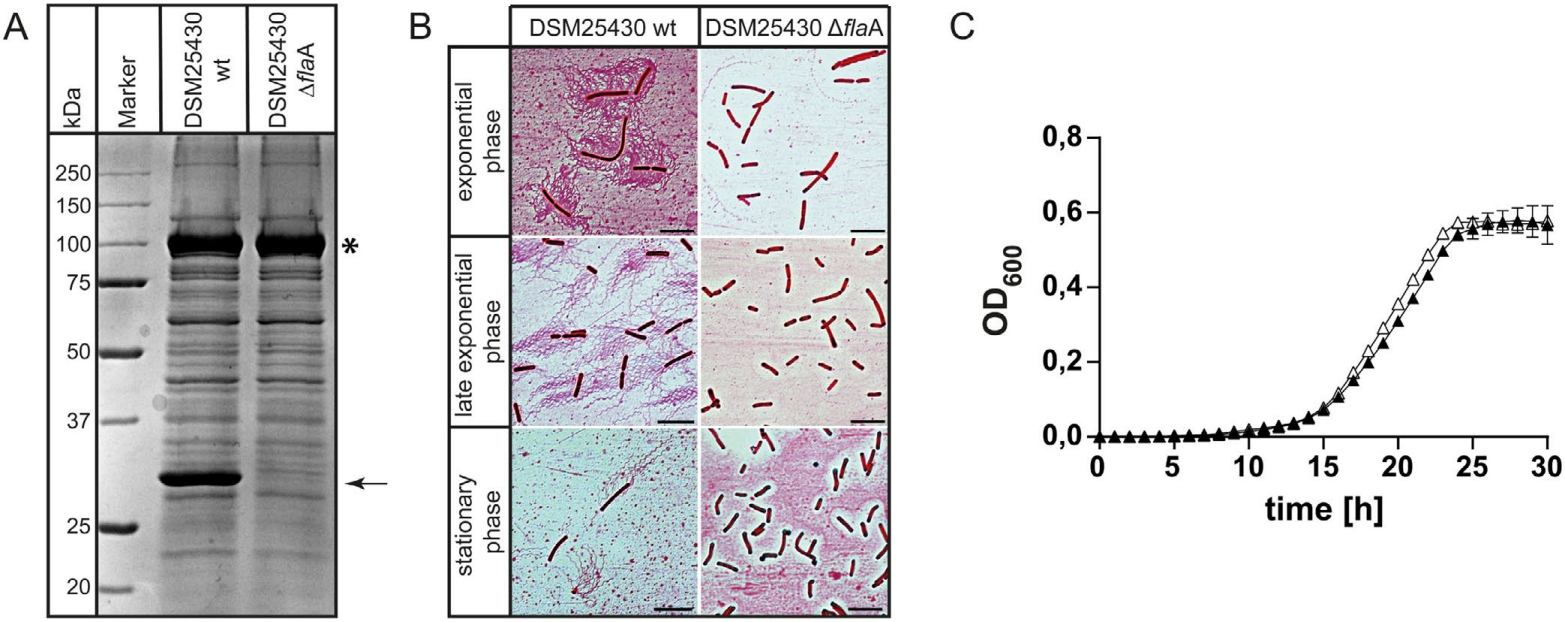
Verification of *fla*A gene inactivation and characterization of the wild‐type and ∆*fla*A mutant strains. (A) SDS‐PAGE analysis of proteins from the *Paenibacillus larvae* gene‐inactivation mutant DSM25430 ∆*fla*A and the corresponding wild type. The protein pattern exhibited a clear difference in the presence of a dominant protein band within the indicated region (black arrow), which corresponds to flagellin FlaA (31.3 kDa), while the other dominant band representing SplA (black asterisk) was unaltered. (B) The utilization of flagella staining revealed the presence of wild‐type *P. larvae* cells with developed flagella at all selected time points. Conversely, the flagellin knockout mutant manifested a smooth surface devoid of filamentous appendages during each growth phase. Scale bars represent 10 μm. (C) Growth curves for *P. larvae* wild type (black triangles) and *fla*A gene inactivation mutant (white triangles) strains were monitored hourly over 30 h under laboratory conditions and revealed no statistically significant differences in growth kinetics (repeated measures two‐way ANOVA with Bonferroni post hoc correction: *p* = 0.0838, *n* = 3). Data represent mean values ± standard deviation (SD) from three biological replicates, each with three technical replicates.

To confirm that *fla*A inactivation in DSM25430 ∆*fla*A resulted not only in the loss of flagellin production (Figure 2A), but indeed in the loss of flagella on the bacterial cell surface, bacterial cells were examined by flagella staining according to Leifson (1951). Because wild‐type bacteria may exist in a flagella‐free state depending on the growth phase (Ferreira et al. 2019; Zhuang et al. 2020), samples were collected at three distinct time points representing the exponential, late exponential and stationary phases. Peritrichous flagella were observed on wild‐type *P. larvae* cells at all three time points, whereas DSM25430 ∆*fla*A lacked detectable cellular appendages throughout the growth curve (Figure 2B). No differences in cell morphology were observed between strains (Figure 2B), and both exhibited virtually identical growth kinetics under laboratory conditions (Figure 2C), indicating that *fla*A inactivation does not cause an intrinsic growth defect.

To exclude unintended mutations or secondary effects associated with the mutagenesis procedure as potential contributors to the mutant phenotype, we performed whole‐genome sequencing. The analysis confirmed that the gene encoding SplA, a cell surface protein involved in cell attachment in *P. larvae* ERIC II (Poppinga et al. 2012), is unaltered in the mutant (Figure S4). Likewise, the paenilarvin gene cluster is intact and unaltered (Figure S5), indicating that paenilarvin biosynthesis and, consequently, its contribution to swarming in *P. larvae* ERIC II (Fünfhaus et al. 2018) are unlikely to be affected. We further confirmed the presence of the TargeTron insert within the *fla*A gene and its absence from all other genomic locations (Figure S6). Whole‐genome alignment of the wild‐type and the mutant *P. larvae* ERIC II strains revealed no additional genomic changes associated with the mutagenesis procedure that could account for the observed mutant phenotype (Figure S6). Together, these results provide strong evidence that the phenotypic differences observed between the wild type and the mutant are attributable to disruption of *fla*A.

### 3.3 Flagella Influence Biofilm Formation of *P. larvae* in Vitro

Flagella play a role in many bacteria as structural components in the formation of biofilms (Haiko and Westerlund‐Wikström 2013; Trunk et al. 2018). To investigate whether the flagella of *P. larvae* are also involved in the recently observed biofilm formation of this bacterial honey bee pathogen (Fünfhaus et al. 2018), the mutant DSM25430 Δ*fla*A was compared with the wild‐type DSM25430 wt in Congo red‐, crystal violet‐ and thioflavin S‐biofilm assays (Figure 3). During static cultivation in liquid medium, DSM25430 wt formed a biofilm, exhibiting a macroscopic structure discernible to the unaided eye (Figure 3A) that was absent in the biofilm formed by DSM25430 Δ*fla*A (Figure 3B). The ECM that maintains the cells within the bacterial community was visualized through the addition of Congo red. In the absence of flagella biosynthesis, the biofilm produced by DSM25430 Δ*fla*A was observed to be more planar and less structured compared to the wild‐type *P. larvae* biofilm (Figure 3D,E). However, the amount of Congo red bound by ECM components did not differ significantly between DSM25430 wt and DSM25430 Δ*fla*A (Figure 3G; unpaired Student's *t* test: *p* = 0.2443), indicating that matrix production is not affected by *fla*A inactivation and that the observed differences in biofilm formation may be attributed to the absence of flagella as a structuring component.

**FIGURE 3:**
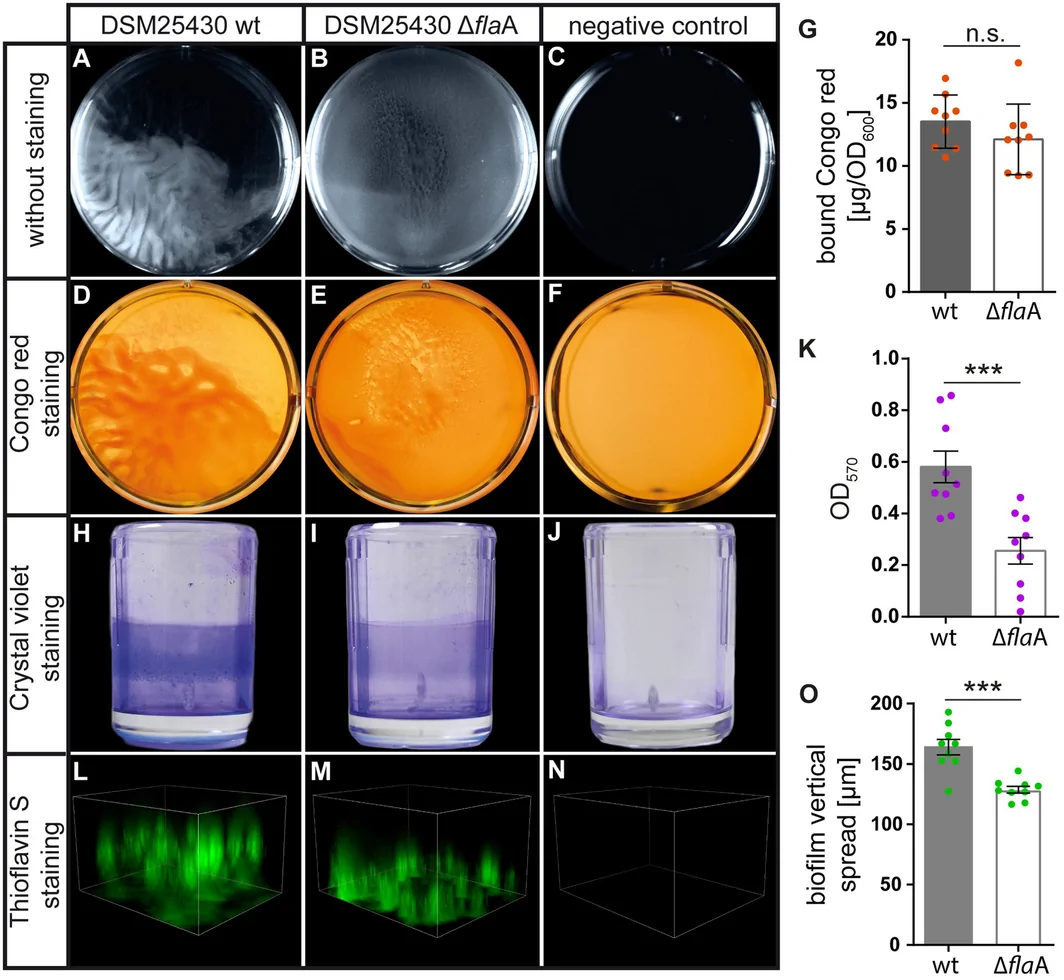
Analysis of biofilm formation of *Paenibacillus larvae* wild type and mutant bacteria. (A–G) Static cultures of wild‐type *P. larvae* ERIC II (DSM25430 wt) and DSM25430 ∆*fla*A were performed in liquid medium (BHI) for 5 days at 37°C without any staining (A and B) followed by a 24 h staining period with the ECM binding dye Congo red (D and E). BHI without inoculated bacteria served as negative controls (C and F). Binding of Congo red by ECM components of the biofilm was quantified and analysed statistically; it did not differ significantly between DSM25430 wt and DSM25430 ∆*fla*A (G; unpaired Student's *t* test: *p* = 0.2443, *n* = 9) despite the obvious structural differences between the biofilms formed by DSM25430 wt (A and D) and DSM25430 ∆*fla*A (B and E). (H–K) After static cultures of *P. larvae* DSM25430 wt and DSM25430 ∆*fla*A in liquid medium (BHI) for 6 days at 37°C, crystal violet staining was used to visualize the *P. larvae* biofilms adhering to the walls of the wells (H and I). Medium without inoculated bacteria served as negative control (J). Crystal violet was quantified and analysed statistically. Stained bacterial biomass differed significantly between DSM25430 wt and DSM25430 ∆*fla*A (K; unpaired Student's *t* test: *p* = 0.0009, *n* = 9). (L–O) Static cultures of *P. larvae* DSM25430 wt and DSM25430 ∆*fla*A were performed in liquid medium (Sf900 SFM II) for 6 days at 37°C in the presence of the amyloid‐binding dye Thioflavin S and thereafter analysed using fluorescence microscopy. Z‐stack processing was performed to obtain three‐dimensional images of the biofilms (L and M). Medium without inoculated bacteria served as negative control (N). While DSM25430 wt predominantly formed floating aggregates, aggregates of DSM25430 ∆*fla*A were observed at the bottom of the wells. Vertical spread of the biofilm was measured and statistically analysed. Vertical spread differed significantly between DSM25430 wt and DSM25430 ∆*fla*A (O; unpaired Student's *t* test: *p* = 0.0001, *n* = 9). Data are shown as scatter plots including mean values ± SD (G) or ± SEM (K and O).

A crystal violet biofilm assay was employed to investigate the potential impact of flagella production on the surface adhesion of *P. larvae* and, consequently, on the formation of surface‐bound biofilms. Crystal violet staining of cells attached to the walls of the cavities of a 96‐well plate, following a static cultivation of bacteria for several days, demonstrated a reduction in the amount of bound biomass formed by DSM25430 Δ*fla*A compared to DSM25430 wt (Figure 3H,I). The quantification of the staining further elucidated that the quantity of adherent biomass formed by *P. larvae* DSM25430 was significantly reduced in the absence of flagella production (Figure 3K; unpaired Student's *t* test: *p* = 0.0009).

To further investigate the effect of the absence of flagella on the structure of the *P. larvae* biofilm, Z‐stack projections were used to generate three‐dimensional images of the wild‐type and mutant *P. larvae* biofilms stained with the fluorescent matrix‐staining dye thioflavin S. After static incubation of *P. larvae* in SF900 SFM II medium, bacterial aggregates of DSM25430 wt appeared mostly in a free‐swimming state (Figure 3L). In contrast, aggregates of DSM25430 Δ*fla*A appeared in contact with the bottom of the wells (Figure 3M), resulting in a significantly reduced vertical spread of the biofilm formed by the bald DSM25430 Δ*fla*A bacteria (Figure 3O; unpaired Student's *t* test: *p* = 0.0001). For all biofilm assays conducted, the respective media without inoculated bacteria served as negative controls, which are shown in Figure 3C,F,J,N.

By testing the flagellin gene‐inactivation mutant of *P. larvae* ERIC II (DSM25430 Δ*fla*A) in the various biofilm assays, we demonstrated that in the absence of flagella production the bacteria were still capable of forming biofilms with an unaltered ECM production. However, the biofilm architecture, surface adhesion and the bacteria's ability to form floating biofilms were impaired by the absence of flagella.

### 3.4 Flagella Are Essential for Swarming Motility of *P. larvae* in Vitro

Another function of flagella is to provide the driving force for the collective movement of bacterial swarms and, hence, flagella are the most crucial prerequisite for swarming (Henrichsen 1972; Kearns 2010). To investigate whether the flagella of *P. larvae* ERIC II play a role in the swarming motility recently described for this bacterial honey bee pathogen (Fünfhaus et al. 2018), we tested DSM25430 wt and the flagellin gene‐inactivation mutant DSM25430 Δ*fla*A on swarm plates consisting of 0.5% BHI agar. As already described, DSM25430 wt was capable of swarming motility and spread collectively as an unstructured swarm on the semisolid agar plates starting from the point of inoculation, reaching the edge of the plates after 3 days of incubation (Fünfhaus et al. 2018). In contrast, DSM25430 Δ*fla*A had completely lost its ability to swarm. Instead of swarming, the mutant cells remained at the inoculation site in the centre of the plates, where they formed a macrocolony (Figure 4).

**FIGURE 4:**
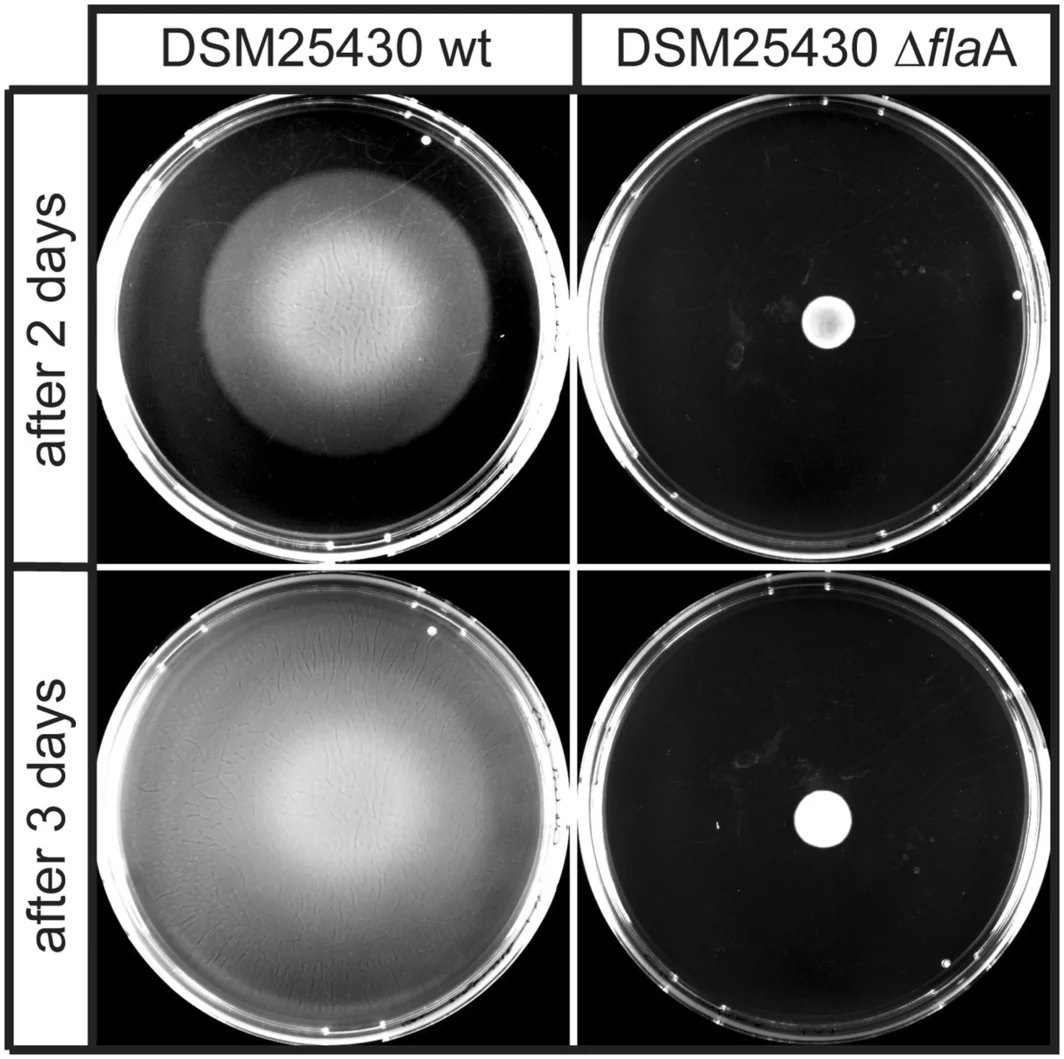
Swarming motility of wild‐type and flagellin‐deficient *Paenibacillus larvae* . Two days after inoculation of 0.5% BHI agar plates with a droplet of bacterial suspensions, *P. larvae* DSM25430 wt demonstrated a shapeless swarm, reaching the edge of the agar plates after 3 days of incubation. In contrast, no swarming behaviour was observed in the flagella‐deficient mutant DSM25430 ∆*fla*A.

### 3.5 Flagella Are Essential for Full Virulence of *P. larvae*

Bacterial flagella, mediating cooperative behaviours like swarming and biofilm formation, are frequently linked to the virulence of pathogenic bacteria (Duan et al. 2013; Haiko and Westerlund‐Wikström 2013). Since the flagella of *P. larvae* ERIC II were both involved in biofilm formation and essential for swarming motility, we hypothesized that they also play a role in the virulence of this *P. larvae* genotype. To test this hypothesis, the survival of honey bee larvae experimentally infected with wild type *P. larvae* DSM25430 wt or the gene‐inactivation mutant for *fla*A, DSM25430 Δ*fla*A, was investigated.

Our results show that by the end of the 14‐day observation period, the Kaplan–Meier estimated survival probability of larvae exposed to spores of *P. larvae* DSM25430 wt was only 17.69%, compared to 37.86% in larvae infected with the gene‐inactivation mutant DSM25430 Δ*fla*A (Figure 5). Survival distributions differed highly significantly over time (log rank (Mantel‐Cox) test: *χ* ^2^ = 11.54, *p* = 0.0007). Calculation of the HR revealed a 69.2% higher relative risk of death (HR_wt/ΔflaA_ of 1.692 (95% CI: 1.24–2.317)) in the group of larvae infected with wild type bacteria compared to the group of larvae infected with the mutant bacteria lacking flagella (Figure 2) and consequently having an impaired ability to form biofilms (Figure 3) and having lost their ability to swarm (Figure 4). Therefore, the flagella of *P. larvae* ERIC II can be considered an important virulence factor of this genotype of *P. larvae* .

**FIGURE 5:**
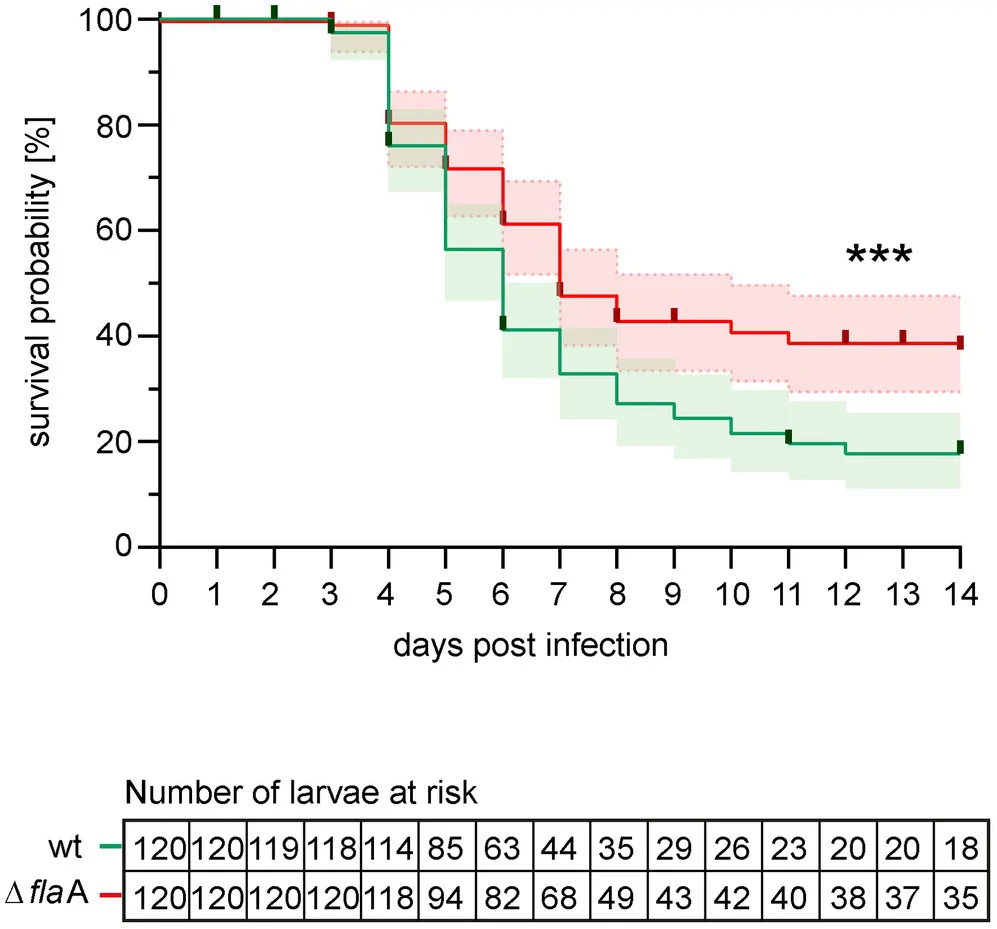
Survival analysis of honey bee larvae exposed to wild‐type *Paenibacillus larvae* or a flagella deficient mutant. Honey bee larvae were infected with wild‐type *P. larvae* (DSM25430 wt; green line) or the corresponding *fla*A inactivation mutant of *P. larvae* (DSM25430 ∆*fla*A; red line). Daily larval mortality due to *P. larvae* infection was recorded over 14 days, revealing a significant difference in survival between groups (log‐rank [Mantel‐Cox] test: *χ* ^2^ = 11.54, *p* = 0.0007, *n* = 120 per group). The hazard ratio (HR_wt/ΔflaA_) was 1.692 (95% CI: 1.24–2.317), indicating an increased risk of death (69.2%) in the wild‐type group compared to the ∆*fla*A mutant group. Vertical tick marks indicate censored observations. The table shows the number of larvae at risk at each time point.

### 3.6 Flagella Are Essential for Multicellular Behaviour of *P. larvae* in the Larval Host

To better understand the role of the *P. larvae* flagella as a virulence factor during pathogenesis in infected larvae, FISH using a *P. larvae* ‐specific, 16S rRNA‐targeted oligonucleotide probe was employed to analyse the multicellular bacterial behaviour in honey bee larvae infected with either *P. larvae* DSM25430 wt or DSM25430 Δ*fla*A. Noninfected larvae served as controls.

As expected, examination of noninfected control larvae via FISH (Figure 6A–D) did not yield any indication of the presence of *P. larvae* . In the cross section of an entire larva (Figure 6A), the foregut, midgut and hindgut, which is not yet connected to the midgut, as well as the midgut epithelium and larval tissues can be seen, but were devoid of any signals for *P. larvae* . Even at higher magnification (Figure 6B–D), no signals for *P. larvae* were detectable.

**FIGURE 6:**
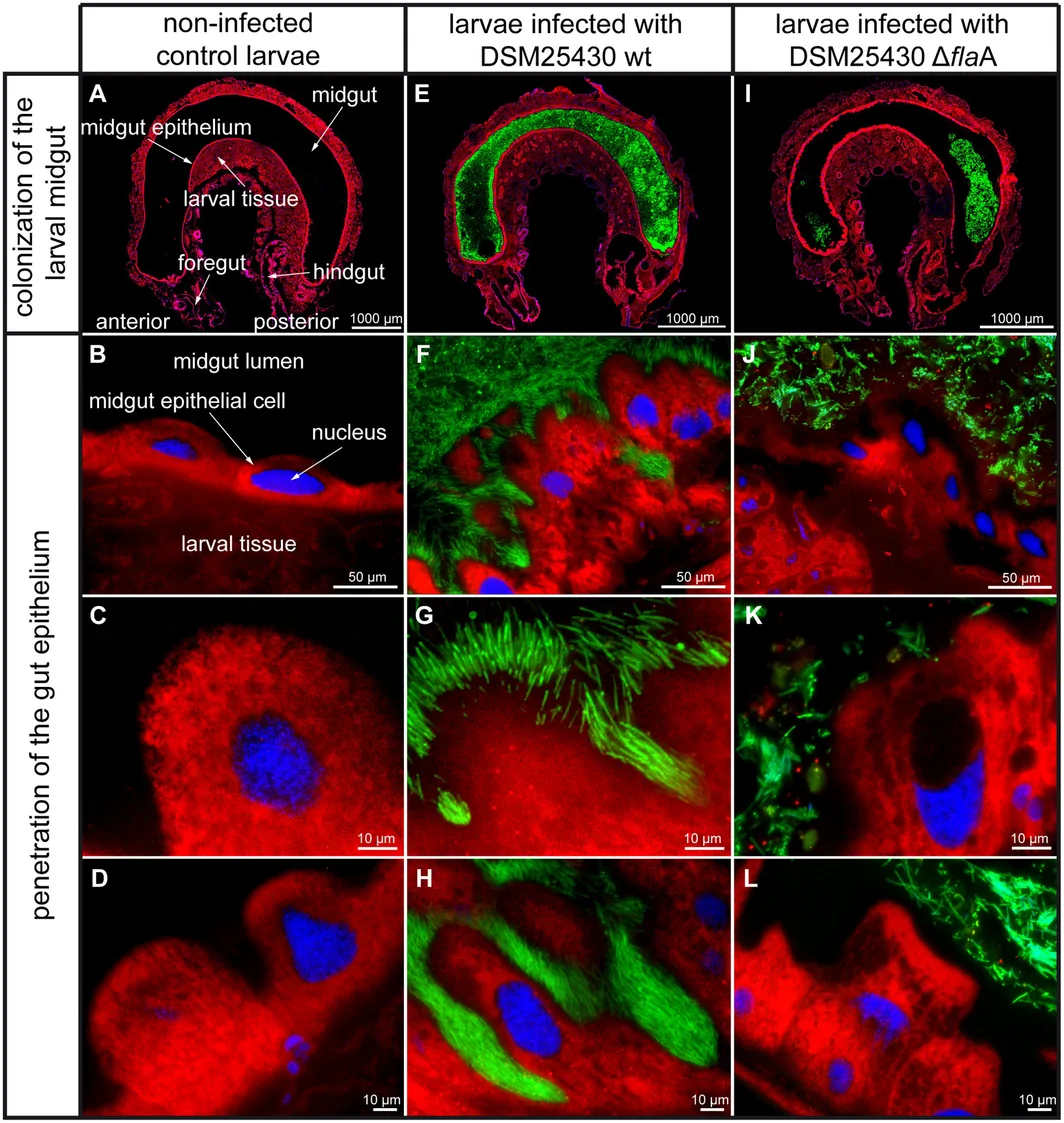
Fluorescence in situ hybridization (FISH) analysis of honey bee larvae infected with wild‐type *Paenibacillus larvae* and the ∆*fla*A mutant. A universal eukaryotic 18S rRNA‐targeted probe (red fluorescence) and a *P. larvae* ‐specific 16S rRNA‐targeted probe (pseudo‐coloured green) were used to analyse sections of noninfected control larvae and larvae infected with *P. larvae* DSM25430 wt or DSM25430 ∆*fla*A. Eukaryotic nuclei were stained with DAPI (blue fluorescence). (A–L) Absence of flagella production was associated with reduced colonization of the larval midgut and impaired penetration of the midgut epithelium. (A) The anterior and posterior ends of the larvae are indicated; the midgut, foregut and hindgut, as well as the midgut epithelium and larval tissues, are marked by arrows. (B) Arrows indicate a midgut epithelial cell and its nucleus. The midgut lumen and larval tissue are indicated. (A, E, I) Representative images of 4‐day‐old live larvae. (B–D, F–H, J–L) Representative images of 5‐day‐old live larvae.

The analysis of live larvae infected with wild‐type *P. larvae* DSM25430 wt (Figure 6E–H) revealed that 4 days postinfection the bacteria had colonized the entire midgut lumen from the anterior to the posterior end (Figure 6E). In the anterior part of the midgut, the bacteria appeared to arrange themselves along the epithelium, forming a sort of ‘lawn’ on the epithelial cell layer. In live larvae 5 days postinfection (Figure 6F–H), the onset of paracellular penetration of the epithelium by the bacteria was clearly visible. The bacteria were observed to accumulate in close proximity to the epithelial layer, and were also visible within the intercellular space between the larval gut cells. At higher magnification (Figure 6G,H), it appeared that the bacteria either aligned themselves in parallel so that their tips attached to the surface of the epithelial cells, thereby forming a ‘lawn’ on the cells, or that they formed three‐dimensional ‘rafts’ of parallel‐aligned bacteria which moved in a clearly coordinated manner in one direction towards the haemocoel, utilizing the intercellular space between neighbouring epithelial cells.

In contrast to the observations in larvae infected with wild‐type *P. larvae* , the bacterial population of live larvae infected with the *fla*A gene‐inactivation mutant DSM25430 Δ*fla*A was repeatedly observed 4 days postinfection only in restricted areas of the midgut (Figure 6I). This suggests that the absence of flagella impaired the ability of *P. larvae* to colonize the gut lumen and establish an infection throughout the larval intestine, consistent with a defect in spatial dissemination rather than a general growth defect (Figure S7, Figure 2C). Moreover, the flagella‐deficient bacteria did not approach the epithelium or form the characteristic ‘lawn’ on the epithelial surface observed in live larvae infected with the wild‐type, flagellated bacteria. At higher magnification (Figure 6J–L), it can be seen that the flagella‐deficient bacteria were scattered and distributed randomly, even when they were in close proximity to the epithelial cells. No coordinated multicellular behaviour was evident and no bacteria had yet entered the intercellular space; hence, there were no signs that the bacteria were attempting to penetrate the epithelium and invade the hemocoel.

The overall findings from our FISH analysis indicate that flagellum‐driven movement through the host was critical for successful colonization of the gut lumen. Furthermore, our results show that the flagella also played a role in *P. larvae* targeting epithelial cells and penetrating the epithelium.

## 4 Discussion

In the past two decades, significant advancements have been made in elucidating the pathogenesis mechanisms of *P. larvae* , which are pivotal for the detection of putative targets for sustainable disease control and curative measures against AFB. However, the number of unanswered questions concerning the pathomechanisms of *P. larvae* remains substantial. We here report on the newly discovered role of flagella in the pathogenesis of *P. larvae* ERIC II infections.

Already at the time of its initial discovery in the early 20th century, *P. larvae* was described as peritrichously flagellated (White 1920). Our *in silico* analysis now revealed that indeed all essential flagella biogenesis genes for the basal body, the hook and the filament are present in the genome of *P. larvae* ERIC II (strain DSM25430; GCF_000511405.1). The genomic organization of the genes into four clusters resembles the organization of the gene clusters for flagellum biosynthesis in *B. subtilis* (Mukherjee and Kearns 2014). Only one open reading frame encoding the filament structural protein flagellin of *P. larvae* (designated *fla*A) was detected in the *P. larvae* genome, hence the filament of *P. larvae* is constructed solely from the product of a single gene, a feature shared with *Pseudomonas aeruginosa* , *B. subtilis* and the majority of *E. coli* species (Wang et al. 2003; Mukherjee and Kearns 2014; Bouteiller et al. 2021). This finding was indirectly supported by the fact that the gene‐inactivation of *fla*A resulted in the complete loss of flagellin production and, consequently, in a flagella‐deficient ‘bald’ phenotype of the *fla*A‐gene‐inactivation mutant *P. larvae* DSM25430 ∆*fla*A.

The synthesis of flagella poses an energy‐expensive process for bacteria (Macnab 1992), and the fact that *P. larvae* expends the energy to form a high number of flagella on the entire cell surface suggests that these cell organelles play a major role for this pathogen during the infection process. Our results now demonstrate that the flagellum functions as a virulence factor for *P. larvae* by contributing to collective and multicellular behaviours like biofilm formation and swarming not only in vitro, but also in vivo.

It is well documented that flagella play a pivotal role in facilitating the adherence process essential to the formation of bound biofilms for multiple bacterial species (O'Toole and Kolter 1998; Lemon et al. 2007; Friedlander et al. 2013; Svensson et al. 2014). The ability of *P. larvae* to form biofilms both adhering to solid surfaces and floating in liquid medium has been recently described (Fünfhaus et al. 2018) and the present study now provides evidence that flagella also play a role in biofilm formation in *P. larvae* . In vitro assays showed that the absence of flagella did not completely impede the formation of a biofilm by *P. larvae* , but resulted (i) in a significant alteration of the biofilm's structural integrity, (ii) in a significantly reduced capability of adhesion to solid, nonbiological surfaces and (iii) in significant changes of the 3D‐structure of the floating biofilms (Figure 3). Based on these observations, we propose that the flagella of *P. larvae* fulfil three distinct functions in the formation of *P. larvae* biofilms: (i) they serve as a structural element, (ii) they facilitate adhesion to surfaces and (iii) they contribute to the buoyancy of floating aggregations in a liquid environment. It is plausible that two of the functions of the flagella—namely their role as structural elements and their contribution to the formation of floating aggregates—are relevant during infection, both while the bacteria proliferate within the larval midgut lumen and subsequently during colonization of the larval cadaver. In contrast, attachment to solid nonbiological surfaces is unlikely to play a role in vivo. Rather, adhesion to biological surfaces and the resulting formation of epithelial biofilms—as has been described for numerous intestinal pathogens (Motta et al. 2021; Wang et al. 2023)—is likely to be the in vivo function of *P. larvae* flagella. And indeed, FISH analysis of larvae infected with *P. larvae* wildtype bacteria revealed the formation of a bacterial ‘lawn’ on the gut epithelial cell layer (Figure 6) composed of bacteria aligned in parallel and adhering to each other with their tips being attached to the surface of the epithelial cells. This organized community of cell surface‐attached *P. larvae* meets the definition of intestinal biofilms (Costerton et al. 1987; Stoodley et al. 2002; Flemming and Wuertz 2019). By showing that the flagellum‐deficient *P. larvae* mutant was no longer able to form this intestinal biofilm in experimentally infected larvae (Figure 6), even when mutant bacteria were present in the direct vicinity of the epithelium, we convincingly linked the flagella of *P. larvae* to *P. larvae* epithelial biofilm formation in vivo. Since the flagella‐deficient *P. larvae* mutant exhibited significantly reduced virulence, we further conclude that biofilm formation on the midgut epithelium is an important step in the pathogenesis of *P. larvae* ERIC II infections.

Recently, the S‐layer protein SplA, a key virulence factor of *P. larvae* ERIC II, has been characterized as a cell adhesion‐mediating protein that plays a critical role in *P. larvae* adhesion to the larval midgut epithelium, which in turn was shown to be a key step in the pathogenesis of *P. larvae* and a prerequisite for the subsequent breaching of the epithelium (Poppinga et al. 2012). Building upon this understanding, we now additionally recognize the critical role of flagella in the adhesion process and extend our understanding by realizing that not only adhesion to the cell surface but the formation of an organized cell surface‐attached *P. larvae* ERIC II biofilm is essential for full virulence of this *P. larvae* genotype.

Another form of collective multicellular behaviour in bacteria, powered by rotating flagella, is swarming motility across semi‐solid media (Henrichsen 1972; Kearns 2010). Many bacterial pathogens employ this flagella‐driven motility to colonize nutrient‐rich environments and host tissues, but also to promote initial surface expansion of bacteria in order to facilitate biofilm formation (Verstraeten et al. 2008; Chaban et al. 2015). Swarming is therefore frequently associated with enhanced virulence in bacterial pathogens ((Kearns 2010; Rütschlin and Böttcher 2020) and references therein).

The ability of *P. larvae* ERIC II to exhibit swarming motility on semi‐solid agar has only recently been described (Fünfhaus et al. 2018). As swarming is a flagella‐driven motility, it was not surprising that in the flagella‐deficient mutant *P. larvae* DSM25430 ∆*fla*A, swarming was completely inhibited in vitro. FISH analysis of larvae from laboratory infection experiments performed with *P. larvae* wildtype bacteria revealed collective, coordinated and directed swarm‐like but three‐dimensional migration of *P. larvae* into the paracellular space for breaching the epithelial barrier (Figure 6). A similar collective bacterial motility could not be observed in larvae infected with the flagella‐deficient mutant DSM25430 Δ*fla*A. Since the flagella‐deficient *P. larvae* mutant exhibited significantly reduced virulence, we further conclude that this swarm‐like but three‐dimensional migration of *P. larvae* into the paracellular space is an important step in the pathogenesis of *P. larvae* ERIC II infections.

The term ‘swarming’ is classically used to describe flagella‐driven collective motility on two‐dimensional (2D) surfaces (Kaiser 2007). In contrast to this 2D‐swarming, several bacteria, like for instance *E. coli* , *P. aeruginosa* and *Serratia marcescens,* have also been observed to display swarm‐like collective movement in three‐dimensional matrices including hydrogels, mucus/slime and porous environments (Chuang et al. 2016; Feng and He 2017; Partridge et al. 2018; Bhattacharjee and Datta 2019). These behaviours are referred to as swarm‐like behaviour, volumetric swarming, multilayer swarming or 3D collective motility, but increasingly also as three‐dimensional (3D) swarming. Here, we use the latter term ‘3D swarming’ to describe the coordinated, flagella‐driven, swarm‐like migration of *P. larvae* ERIC II into the paracellular space when attempting to breach the midgut epithelium. The reduction in virulence was accompanied by the loss of coordinated 3D swarming motility, indicating an important role of flagella‐mediated 3D swarming in *P. larvae* virulence in vivo.

FISH analyses of larvae obtained from laboratory infection experiments further suggested that 3D swarming originated from the biofilms formed on the epithelial surface. Thus, the initially rather stationary 2D biofilm appeared to transition into a motile multicellular structure with a pronounced 3D architecture. Such a transition likely depends on the continued synthesis and functionality of flagella during biofilm maturation, a phenomenon that has previously been described in several bacterial species. In *P. aeruginosa* , it has been demonstrated that the flagella formed scaffold‐like structures, thereby providing the biofilm with mechanical and physical strength (Ozer et al. 2021). In the lower layers of the biofilm macrocolonies of *E. coli* , the cells could be observed in a tight network of interwoven flagella, which were required for the complete development of the biofilm structure (Serra et al. 2013). We propose that the formation of such a scaffold by interwaving flagella provides the *P. larvae* biofilm with the necessary strength to persist in the host environment, while at the same time allowing the transition into the state of 3D swarming.

The observed 3D swarming behaviour raises the hypothesis that *P. larvae* ERIC II utilizes swarming behaviour to generate sufficient force to penetrate the intercellular spaces and overcome the cell‐to‐cell connections presumably with the additional help of the numerous proteases secreted by *P. larvae* (Holst and Sturtevant 1940; Dancer and Chantawannakul 1997; Antunez et al. 2009, 2011). Further research is needed to elucidate whether the differentiation into a swarming phenotype is accompanied by an increased expression of the pathogen's virulence factors, for example, proteases that facilitate the movement of *P. larvae* ERIC II through the paracellular space. The increased expression of virulence genes during swarming behaviour has been previously observed in *Proteus mirabilis* and *P. aeruginosa* (Allison et al. 1992; Overhage et al. 2008).

The virulent, but nonswarming *P. larvae* genotype ERIC I (Fünfhaus et al. 2018), which also lacks cell adhesion (Poppinga et al. 2012), has previously been reported to produce toxins targeting epithelial cells (Fünfhaus et al. 2013; Ebeling et al. 2017, 2019, 2021). A toxin‐mediated pathogenesis strategy that enables epithelial damage from a distance may allow bacterial passage through the epithelium without the necessity for epithelial attachment, biofilm formation and subsequent 3D swarming. In contrast, the pathogenesis strategy of *P. larvae* ERIC II appears to be strongly dependent on flagella, which contribute to midgut colonization, epithelial adhesion, biofilm formation on the epithelium and subsequent penetration of the intercellular space via three‐dimensional swarm‐like motility (3D swarming). Taken together, our data suggest that flagellar production is critical for successful completion of the infection cycle in *P. larvae* ERIC II and thus for subsequent transmission to naïve larval hosts. These findings significantly deepen our understanding of the molecular mechanisms underlying *P. larvae* pathogenesis and provide a framework for future studies aimed at improving strategies for the control and eradication of AFB.

## 5 Conclusion

Our findings identify the peritrichous flagella of *P. larvae* ERIC II as important factors in pathogenesis. Loss of flagella production in vitro not only abolishes swarming motility but also impairs biofilm formation, resulting in reduced adhesion, altered biofilm architecture and the loss of the characteristic floating phenotype. Consequently, the flagella‐deficient mutant exhibits significantly reduced virulence towards honey bee larvae in vivo. Combined with our FISH analyses of infected larvae, these results demonstrate that flagella contribute to multiple stages of the infection process, including gut colonization, intestinal biofilm formation, midgut epithelial invasion via 3D swarming and dissemination within the host. We therefore propose that flagella‐mediated motility and multicellular behaviour are central mechanisms enabling *P. larvae* to efficiently exploit the larval host, maximize bacterial biomass production and sporulation and ultimately ensure transmission. These findings establish flagella as central virulence determinants of *P. larvae* ERIC II and significantly advance our understanding of the mechanisms underlying AFB pathogenesis, and provide a basis for the development of novel disease‐control strategies.

## Author Contributions

**Josefine Göbel:** conceptualization, methodology, investigation, formal analysis, writing – original draft, writing – review and editing. **Anne Fünfhaus:** methodology, investigation, supervision, writing – review and editing. **Elke Genersch:** conceptualization, methodology, supervision, funding acquisition, formal analysis, writing – original draft, writing – review and editing.

## Funding

This work was supported by the Ministries Responsible for Agriculture of the German Federal States of Brandenburg, Saxony‐Anhalt, Saxony, and the Senate of Berlin, Germany.

## Ethics Statement

The authors declare that all prevailing local, national and international regulations and conventions, and normal scientific ethical practices have been respected.

## Conflicts of Interest

The authors declare no conflicts of interest.

## Supporting information


**Table S1:** Description of flagella biosynthesis genes in *P. larvae* ERIC II (DSM25430).
**Figure S1:** Verification of gene inactivation of *P. larvae* knockout mutant. PCR analysis was used to confirm the integration of the approximately 900 bp Ll.LtrB intron into the target gene *fla*A. For this purpose, primers flanking the insertion site of the intron were used. The anticipated amplicon sizes for the knockout PCR were 825 bp in the absence of an insertion and 1740 bp in the event of a successful insertion.
**Figure S2:** Full‐height image of SDS‐PAGE represented in Figure 2. The image was cropped only laterally and shows full vertical extent of the corresponding lanes. The position of the flagellin protein FlaA is indicated by a black arrow. The surface layer protein SplA is indicated by an asterisk.
**Figure S3:** Rearing of honey bee larvae in the laboratory. In order to test the virulence of the constructed *P. larvae* knockout mutant, in vivo infection assays were carried out with bee larvae. The rearing of approximately 12‐h‐old larvae was carried out in the laboratory in 24‐well plates at 35°C. The larvae were transferred daily to fresh food until the onset of defecation, after which the larvae were transferred to paper towel‐lined wells. For infection with *P. larvae* , spores were added to the food for the first 24 h of the experiment. The images depict healthy larvae from a naive control group on Days 0–14. Scale bars correspond to 2000 μm.
**Figure S4:** Alignment of *spl*A in wild‐type *P. larvae* and the *P. larvae* Δ*fla*A mutant. The *spl*A gene was not affected during construction of the *fla*A mutant. The genomic sequence of *P. larvae* *spl*A was 100% identical between DSM25430 wt (1) and DSM25430 Δ*fla*A (2). The uninterrupted green bar above the alignment indicates 100% sequence identity between the aligned sequences.
**Figure S5:** Paenilarvin (Iturin) gene cluster in wild‐type *P. larvae* and the corresponding flagellin gene inactivation mutant. The construction of the *P. larvae* Δ*fla*A mutant did not affect the paenilarvin gene cluster responsible for the nonribosomal biosynthesis of paenilarvin in *P. larvae* ERIC II. The genomic sequences of the clusters showed 100% identity between DSM25430 wt and DSM25430 Δ*fla*A. Numbers indicate the position at the beginning and end of the cluster according to the NCBI reference sequence NC_023134.1.
**Figure S6:** Whole‐genome Mauve alignment of wild‐type *P. larvae* ERIC II (DSM25430 wt) and the flagellin mutant (DSM25430 Δ*fla*A) genomes. Whole‐genome sequence analysis confirmed that the TargeTron intron was integrated exclusively in the *fla*A gene during mutant construction (asterisk). Additional genomic differences, indicated by red lines, were attributable to transposon insertion events and did not affect any open reading frames. Numbers indicate genomic positions. Whole‐genome sequencing data for *P. larvae* DSM25430 Δ*fla*A were deposited at NCBI under the GenBank accession number JCFKJT000000000.1 (NCBI BioProject: PRJNA1516485).
**Figure S7:** Proliferation of wild‐type *P. larvae* and the *P. larvae* Δ*fla*A mutant in the gut lumen of honey bee larvae. A *P. larvae* ‐specific 16S rRNA‐targeted probe (pseudo‐coloured green) was used to visualize vegetative *P. larvae* in the midgut of larvae infected with DSM25430 wt or DSM25430 Δ*fla*A. Larval tissue was visualized with a universal eukaryotic 18S rRNA‐targeted probe (red fluorescence). Eukaryotic nuclei were stained with DAPI (blue fluorescence).

## Data Availability

The authors declare that all data generated during this study are included in this article (and its Supporting Information). The genome sequence data of the gene‐inactivation mutant constructed in this study have been deposited at NCBI as BioProject PRJNA1516485 and GenBank accession number JCFKJT000000000.1 (the master record for the whole genome shotgun sequencing project). All other relevant raw data relating to the article are available from the corresponding author (E.G.) upon reasonable request.
