# Supplementary material for: Flagella‐Mediated Biofilm Formation and 3D Swarming Drive Virulence in the Honey Bee Pathogen Paenibacillus larvae
Source: Environ Microbiol. 2026 Nov 3;28(11):e70477. doi: 10.1111/1462-2920.70477 (PMC13731065; doi:10.1111/1462-2920.70477)
Supplement: Supplementary file 1 [file EMI-28-e70477-s001.docx]

**Supplementary material for:**

**Flagella-mediated biofilm formation and 3D swarming drive virulence in the honey bee pathogen *Paenibacillus larvae***

Josefine Göbel^1^, Anne Fünfhaus^1^, Elke Genersch^1,2*^

^1^ Institute for Bee Research, Department of Molecular Microbiology and Bee Diseases, Hohen Neuendorf, Germany

^2^Freie Universität Berlin, Department of Veterinary Medicine, Institute of Microbiology and Epizootics, Berlin, Germany

*Corresponding author:

Elke Genersch, Institute for Bee Research, Friedrich-Engels-Str. 32, 16540 Hohen Neuendorf, Germany

fon: ++49 (0)3303 293833

fax: ++49 (0)3303 293840

E-mail: [elke.genersch@hu-berlin.de](mailto:elke.genersch@hu-berlin.de)

**Supplementary Tables and Figures**

**Table S1: Description of flagella biosynthesis genes in *P. larvae* ERIC II (DSM25430).**

| **Gene** | **Gene lenght  (bp)** | **Protein mass (kDa)** | **Putative protein product** |
| --- | --- | --- | --- |
| *flh*O | 894 | 33.2 | Flagellar basal body protein FlhO |
| *flh*P | 828 | 30 | Flagellar basal body protein FlhP |
|  |  |  |  |
| *yvy*F | 480 | 18.4 | putative transcriptional regulator |
| *flg*M | 261 | 9.6 | Flagella biosynthesis anti-sigma factor FlgM |
| *flg*N | 504 | 19.4 | Flagellar protein FlgN |
| *flg*K | 1560 | 57 | Flagellar hook-associated protein FlgK |
| *flg*L | 900 | 33.8 | Flagellar hook-associated protein FlgL |
| *yvi*E | 561 | 21.1 | uncharacterized protein |
| *fli*W | 462 | 17.5 | Flagellar assembly factor FliW |
| *csr*A | 282 | 10.5 | Carbon storage regulator CsrA |
| *fla*A | 858 | 31.3 | Flagellin |
| *fli*D | 1518 | 55.3 | Flagella filament capping protein FliD |
| *fli*S | 399 | 14.8 | Flagellar export chaperone FliS |
| *fli*T | 354 | 13.4 | Flagellar protein FliT |
|  |  |  |  |
| *mot*B | 669 | 24.7 | Flagellar motor protein MotB |
| *mot*A | 798 | 28.2 | Flagellar motor protein MotA |
|  |  |  |  |
| fapA | 1404 | 50.9 | Flagellar assembly protein A |
| *sigD* | 789 | 30.4 | RNA polymerase sigma-D factor |
| n/a | 435 | 16.2 | hypothetical protein |
| *che*D | 495 | 17.3 | Chemotaxis protein CheD |
| *che*C | 618 | 21.8 | Chemotaxis protein CheC |
| *che*W | 462 | 17 | Chemotaxis protein CheW |
| *che*A | 2061 | 75.1 | Chemotaxisprotein CheA |
| *che*B | 1143 | 40.9 | Chemotaxis response regulator protein-glutamate methylesterase CheB |
| *flh*G | 879 | 32.7 | Flagellar synthesis regulator FlhG |
| *flh*F | 1398 | 51.1 | Flagellar biosynthesis protein FlhF |
| *flh*A | 2034 | 74.2 | Flagellar biosynthesis protein FlhA |
| *flh*B | 1092 | 41 | Flagellar biosynthetic protein FlhB |
| *fli*R | 789 | 29 | Flagellar biosynthetic protein FliR |
| *fli*Q | 270 | 9.8 | Flagellar biosynthesis protein FliQ |
| *fli*P | 636 | 23.3 | Flagellar biosynthetic protein FliP |
| *fli*O | 642 | 23.8 | Flagellar biosynthetic protein fliO |
| *che*Y | 363 | 13.2 | Response regulator CheY |
| *fli*Y | 1203 | 43.2 | Flagellar motor switch phosphatase FliY |
| *fli*M | 999 | 37.6 | Flagellar motor switch protein FliM |
| *fli*L | 453 | 16.5 | Flagellar basal body-associated FliL-family protein |
| *swrD* | 222 | 7.9 | Swarming motility Protein D |
| *flg*G | 813 | 28.9 | Flagellar basal-body rod protein FlgG |
| *flg*E | 390 | 14.3 | Flagellar hook protein |
| *flg*D | 573 | 20.8 | Flagellar hook capping protein FlgD |
| *fli*K | 1404 | 50.6 | Flagellar hook-length control protein FliK |
| *ylx*F | 912 | 33.4 | Magnesium transporter MgtE N-terminal domain-containing protein |
| *fli*J | 444 | 17.6 | Flagellar export protein FliJ |
| *fli*I | 1320 | 47.6 | Flagellar protein export ATPase FliI |
| *fli*H | 819 | 31.1 | Flagellar assembly protein FliH |
| *fli*G | 1017 | 37.8 | Flagellar motor switch protein FliG |
| *fli*F | 1584 | 57.8 | Flagellar basal-body MS-ring/collar protein FliF |
| *fli*E | 315 | 11.6 | Flagellar hook-basal body complex protein FliE |
| *flg*C | 450 | 16.5 | Flagellar basal body rod protein FlgC |
| *flg*B | 414 | 15.6 | Flagellar basal body rod protein FlgB |

**Figure S1: Verification of gene inactivation of *P. larvae* knockout mutant.** PCR analysis was used to confirm the integration of the approximately 900 bp Ll.LtrB intron into the target gene *fla*A. For this purpose, primers flanking the insertion site of the intron were used. The anticipated amplicon sizes for the knockout PCR were 825 bp in the absence of an insertion and 1,740 bp in the event of a successful insertion.

**Figure S2: Full-height image of SDS-PAGE represented in Figure 2.** The image was cropped only laterally and shows full vertical extent of the corresponding lanes. The position of the flagellin protein FlaA is indicated by a black arrow. The surface layer protein SplA is indicated by an asterisk.

**Figure S3: Rearing of honey bee larvae in the laboratory.** In order to test the virulence of the constructed *P. larvae* knockout mutant, *in vivo* infection assays were carried out with bee larvae. The rearing of approximately 12-hour-old larvae was carried out in the laboratory in 24-well plates at 35 °C. The larvae were transferred daily to fresh food until the onset of defecation, after which the larvae were transferred to paper towel-lined wells. For infection with *P. larvae*, spores were added to the food for the first 24 hours of the experiment. The images depict healthy larvae from a naive control group on days 0 to 14. Scale bars correspond to 2000 µm.

**Figure S4: Alignment of *spl*A in wild-type *P. larvae* and the *P. larvae* Δ*fla*A mutant.** The *spl*A gene was not affected during construction of the *fla*A mutant. The genomic sequence of *P. larvae* *spl*A was 100 % identical between DSM25430 wt (1) and DSM25430 Δ*fla*A (2). The uninterrupted green bar above the alignment indicates 100 % sequence identity between the aligned sequences.

**Figure S5: Paenilarvin (Iturin) gene cluster in wild-type *P. larvae* and the corresponding flagellin gene inactivation mutant.** The construction of the *P. larvae* Δ*fla*A mutant did not affect the paenilarvin gene cluster responsible for the non-ribosomal biosynthesis of paenilarvin in *P. larvae* ERIC II. The genomic sequences of the clusters showed 100 % identity between DSM25430 wt and DSM25430 Δ*fla*A. Numbers indicate the position at the beginning and end of the cluster according to the NCBI reference sequence NC_023134.1.

**Figure S6: Whole-genome Mauve alignment of wild-type *P. larvae* ERIC II (DSM25430 wt) and the flagellin mutant (DSM25430 Δ*fla*A) genomes.** Whole-genome sequence analysis confirmed that the TargeTron intron was integrated exclusively in the *fla*A gene during mutant construction (asterisk). Additional genomic differences, indicated by red lines, were attributable to transposon insertion events and did not affect any open reading frames. Numbers indicate genomic positions. Whole-genome sequencing data for *P. larvae* DSM25430 Δ*fla*A have been deposited at NCBI as BioProject PRJNA1516485 and GenBank accession number JCFKJT000000000.1 (the master record for the whole genome shotgun sequencing project).

**Figure S7: Proliferation of wild-type *P. larvae* and the *P. larvae* Δ*fla*A mutant in the gut lumen of honey bee larvae.** A *P. larvae*-specific 16S rRNA-targeted probe (pseudo-coloured green) was used to visualize vegetative *P. larvae* in the midgut of larvae infected with DSM25430 wt or DSM25430 Δ*fla*A. Larval tissue was visualized with a universal eukaryotic 18S rRNA-targeted probe (red fluorescence). Eukaryotic nuclei were stained with DAPI (blue fluorescence).
